# Refining the Predictive Accuracy of Membranous Urethral Length for Post-Prostatectomy Incontinence: A Standardized Approach in the Korean Population

**DOI:** 10.3390/jcm15124454

**Published:** 2026-06-09

**Authors:** Jong Kyou Kwon, Do Kyung Kim, Jin hyung Jeon, Sungun Bang, Kyo Chul Koo, Kwang Suk Lee, Eun-Suk Cho, Kang Su Cho

**Affiliations:** 1 Department of Urology, Prostate Cancer Center, Gangnam Severance Hospital, Yonsei University College of Medicine, Seoul 06273, Republic of Korea; jkstorm@yuhs.ac (J.K.K.); dokyung80@yuhs.ac (D.K.K.); bbsungun@yuhs.ac (S.B.); gckoo@yuhs.ac (K.C.K.); calmenow@yuhs.ac (K.S.L.); 2 Department of Urology, Yong-In Severance Hospital, Yonsei University College of Medicine, Yong-In 17046, Republic of Korea; jun1644@yuhs.ac; 3 Department of Radiology, Gangnam Severance Hospital, Yonsei University College of Medicine, Seoul 06273, Republic of Korea; jjondol@yuhs.ac; 4 Center of Evidence Based Medicine, Institute of Convergence Science, Yonsei University College of Medicine, Seoul 06273, Republic of Korea

**Keywords:** urinary incontinence, prostatectomy, magnetic resonance imaging, urethra, robotic surgical procedures

## Abstract

**Background/Objectives:** Preoperative membranous urethral length (MUL) is a predictor of post-prostatectomy urinary incontinence. However, measurement inconsistencies have hindered the establishment of ethnically specific clinical thresholds. We identified precise and internally validated MUL cutoff values for persistent incontinence at 6 and 12 months in a Korean cohort using a standardized measurement protocol. **Methods:** We retrospectively analyzed 151 patients who underwent robot-assisted radical prostatectomy (RARP) between 2022 and 2024. Preoperative MUL was measured using a 3-axis cross-reference system (CRS) on 3.0T mpMRI. Continence was defined as 0 or 1 safety pad per day. Independent predictors were identified via multivariable logistic regression, and the optimal cutoff values were determined using the Youden index with 1000-iteration bootstrap validation. **Results:** Preoperative MUL was significantly longer in continent than in incontinent patients at 6 (16.7 vs. 13.7 mm) and 12 months (16.7 vs. 11.3 mm; both *p* < 0.001). In the multivariable analysis, MUL was the only significant independent predictor for persistent incontinence (6 months: OR 0.798, *p* < 0.001; 12 months: OR 0.586, *p* < 0.001). The univariable AUROCs for predicting persistent incontinence were 0.707 (6 months) and 0.875 (12 months), whereas the multivariable AUROCs were 0.756 and 0.883, respectively. Optimal cutoff values from bootstrap were 14.00 mm (95% confidence interval [CI], 11.43–16.68) for 6-month and 12.60 mm (95% CI, 11.43–14.37) for 12-month persistent incontinence. **Conclusions:** Using a standardized CRS protocol, this study provides validated population-specific MUL thresholds for predicting persistent incontinence after RARP in Korean men, offering a pragmatic framework for preoperative risk stratification and evidence-based patient counseling.

## 1. Introduction

Robot-assisted radical prostatectomy (RARP) has emerged as a standard of care, offering superior oncological and functional outcomes [1,2,3]. However, post-prostatectomy urinary incontinence (PPI) remains a critical challenge that significantly impairs patients’ quality of life and causes substantial psychological distress [4,5,6,7]. As patients increasingly weigh various curative options, including radiotherapy and particle beam therapy, the speed and quality of persistent incontinence have become decisive factors in treatment selection [8,9,10,11].

Among the various predictors, preoperative membranous urethral length (MUL) is recognized as one of the most significant anatomical determinants of PPI recovery [12,13,14,15,16]. Despite its clinical importance, existing literature on MUL is characterized by significant methodological inconsistencies. Previous studies employed highly variable measurement protocols, ranging from simple sagittal views to inconsistent coronal plane assessments, often without a clear definition of anatomical boundaries. Considerable variability has been reported across previous studies, with some measuring MUL in the sagittal plane, others in the coronal plane, and some using a combination of both; in certain cases, the measurement methodology was not clearly defined [12,14,15,16,17,18,19,20]. This “methodological chaos” led to widely varying results, making it difficult to establish a universally applicable, ethnicity-specific clinical threshold.

To address these limitations, we established and validated a standardized MUL measurement protocol using a cross-linked referencing system to ensure anatomical precision and reproducibility [21]. Although methodological standardization is the first step, applying this rigorous approach to specific populations is essential for clinical decision-making. Recent evidence has highlighted significant racial variations in pelvic anatomy; for instance, Asian men have been reported to have a shorter mean MUL than non-Asian men, which is independently associated with worse urinary function scores following RARP [22]. These anatomical disparities emphasize the need for population-specific brain templates or ethnically specific MUL benchmarks. However, high-quality data focusing on the Korean population using standardized and reproducible metrics are lacking.

In this study, we aimed to transition from methodological standardization to clinical application. Using our previously validated standardized protocol, we analyzed the impact of preoperative MUL on persistent incontinence at 6 and 12 months post-RARP in a Korean cohort. Our objective was not only to confirm this association but also to provide precise, validated clinical cutoff values that can serve as reliable benchmarks for preoperative counseling and surgical planning.

## 2. Materials and Methods

### 2.1. Study Population and Ethical Statement

This retrospective study was conducted according to the ethical principles of the Declaration of Helsinki (2013). The study protocol was approved by the Institutional Review Board (Approval No. 3-2025-0415).

A total of 211 consecutive patients who underwent RARP with preoperative prostate MRI between 2022 and 2024 were initially screened. To ensure the consistency of anatomical measurements, patients who underwent preoperative multiparametric magnetic resonance imaging (mpMRI) at external institutions using heterogeneous protocols were excluded. After further excluding patients with incomplete clinical or follow-up data, as well as those who received adjuvant or salvage radiotherapy and/or androgen deprivation therapy during the follow-up period, the final cohort consisted of 151 patients.

### 2.2. MRI Acquisition Protocol

Preoperative mpMRI was performed using a 3.0 Tesla system (Intera Achieva 3.0 T; Philips Medical Systems, Best, The Netherlands) with a six-channel phased-array coil. Standardized protocol included T2-weighted turbo spin-echo sequences in axial, sagittal, and coronal planes. All datasets were acquired at identical slice locations with a slice thickness of 3 mm and no interslice gaps to minimize the risk of missing the prostatic apex or penile bulb landmarks. Diffusion-weighted imaging and dynamic contrast-enhanced MRI were also used for tumor localization and PI-RADS (v2.1) grading [23].

### 2.3. Standardized MUL Measurement

To address the inherent variability and lack of reproducibility of conventional measurements, preoperative MUL was assessed based on a standardized measurement protocol utilizing a 3-axis cross-reference system (CRS), as previously validated by Kwon et al. [21].

**Figure 1:**
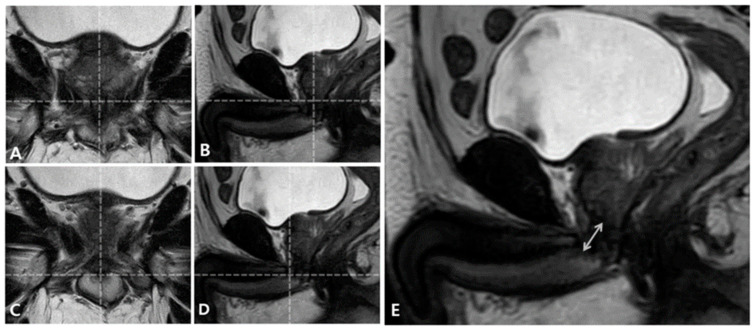
The membranous urethral length (MUL) measurement in prostate MRI. MUL is measured using a 3-axis cross-reference tool on prostate MRI and is defined as the distance from the superior border of the penile bulb to the inferior border of the prostate apex. (**A**,**B**) Identification of the inferior border of the prostate apex in the coronal and mid-sagittal planes. (**C**,**D**) Identification of the superior border of the penile bulb in the coronal and midsagittal planes. (**E**) Visual representation of the complete MUL trajectory across imaging planes. The white double-headed arrow indicates the measured membranous urethral length (MUL).

MUL was defined as the distance from the superior border of the penile bulb to the inferior border of the prostatic apex. Measurements were performed using the CRS tool within the Picture Archiving and Communication System, allowing simultaneous synchronization of coordinates across the sagittal, coronal, and axial planes (Figure 1). This method ensures that anatomical landmarks identified in the sagittal plane are verified in the coronal and axial views, thereby mitigating errors caused by pelvic floor tilting or asymmetrical prostatic apical shapes.

Depending on the individual curvature of the urethra and anatomical variations in the prostate apex, the four to ten parasagittal planes were analyzed alongside the midsagittal plane. This multiplanar approach was employed to precisely capture the shortest functional length of the membranous urethra while accounting for potential distortions in single-plane visualization.

### 2.4. Outcome Definition and Variables

The primary outcome was persistent incontinence of urinary continence at 6 and 12 months postoperatively. Continence was defined as being completely pad-free or using no more than one pad per 24-h period. Clinical variables, including age, body mass index (BMI), preoperative prostate-specific antigen (PSA) levels, prostate volume, underlying comorbidities (hypertension and diabetes mellitus), and perioperative and pathological factors, such as estimated blood loss, seminal vesicle invasion (SVI), extracapsular invasion, positive surgical margin, Gleason score, lymph node dissection (LND), nerve-sparing status (NS), and operative time, were extracted from medical records. Preoperative urinary continence was clinically verified through detailed physician consultation, and patients with pre-existing incontinence were excluded from the cohort. Postoperative urinary continence was evaluated at each outpatient visit. The daily pad usage was explicitly assessed and charted by a dedicated outpatient nurse, followed by confirmation by the attending urologist.

All surgical procedures were performed using a standardized transperitoneal approach by two highly experienced urologists, each possessing over 10 years of surgical expertise in robotic prostatectomy. Additionally, the study cohort included patients who underwent an extended pelvic lymph node dissection (ePLND), which encompassed the removal of the obturator, external iliac, and internal iliac lymph nodes.

### 2.5. Statistical Analysis

Continuous variables are presented as means with standard deviations or medians with ranges, whereas categorical variables are expressed as frequencies and percentages. To further evaluate the functional impact of MUL, patients were stratified into four quartiles (Q1–Q4) based on their preoperative MUL measurements. The clinical, pathological, and operative parameters across these quartiles were compared to assess potential confounding factors. Additionally, the anatomical relationship between prostate volume and MUL was evaluated using Pearson’s correlation coefficient and visualized using a scatter plot. Univariable and multivariable logistic regression analyses were performed to identify independent PPI predictors. Variables with a *p*-value < 0.1 in the univariate analysis (LND, SVI, and operative time) were included in the multivariable model. Age was also intentionally incorporated into the multivariable analysis despite its lack of statistical significance in the univariate model, given its well-documented clinical relevance and reported association with persistent incontinence in the existing literature [24,25].

ROC analysis was used to determine the discriminative ability of MUL, and the optimal cutoff values were calculated using the Youden index. To assess the internal validity and stability of these thresholds, bootstrap analysis with 1000 resamples was performed. All statistical analyses were conducted using R version 4.4.3 (28 February 2025 ucrt), and a *p*-value < 0.05 was considered statistically significant.

## 3. Results

### 3.1. Baseline Characteristics

In total, 151 patients who underwent RARP were included in the final analysis. Baseline clinical and perioperative characteristics stratified by continence status at 6 and 12 months are summarized in Table 1. Six months postoperatively, 109 patients (72.2%) recovered continence, whereas 42 patients (27.8%) remained incontinent. By 12 months postoperatively, the recovery rate improved, with 127 patients (84.1%) achieving continence and 24 patients (15.9%) remaining incontinent. Notably, preoperative MUL was significantly longer in the continence group compared with the incontinence group at both 6 months (16.7 ± 3.7 mm vs. 13.7 ± 4.4 mm, *p* < 0.001) and 12 months (16.7 ± 3.7 mm vs. 11.3 ± 3.1 mm, *p* < 0.001). In addition to the anatomical metrics, several perioperative and pathological factors were significantly different between the two groups. Specifically, at 6 months, the incontinence group demonstrated a significantly higher prevalence of seminal vesicle invasion than the continence recovery group (31.0% vs. 11.0%, *p* = 0.007). Furthermore, the requirement for bilateral lymph node dissection was markedly more frequent in incontinent patients at both 6 (40.5% vs. 6.4%, *p* < 0.001) and 12 months (45.8% vs. 10.2%, *p* = 0.001). Regarding operative outcomes, the mean operative time was significantly longer in the incontinence group at 6 months (176.7 ± 38.9 min vs. 155.0 ± 37.4 min, *p* = 0.003). Conversely, other clinical parameters including estimated blood loss (225.4 ± 174.6 mL for the total cohort), the presence of comorbidities such as hypertension or diabetes, and the status of surgical margins did not exhibit statistically significant differences according to persistent incontinence at either time point. Other variables, including age, PSA level, and BMI, did not differ significantly between the two groups. Furthermore, the postoperative urinary continence rates demonstrated a stepwise improvement across the preoperative MUL quartiles (Table 2).

### 3.2. Multivariable Logistic Regression Analysis

In the univariable logistic regression analysis, operative time (*p* = 0.003), seminal vesicle invasion (*p* = 0.004), bilateral lymph node dissection (*p* < 0.001), and MUL (*p* < 0.001) were significant predictors of persistent incontinence at 6 months. At 12 months, bilateral lymph node dissection (*p* < 0.001) and MUL (*p* < 0.001) remained significant. Following these findings, multivariable logistic regression analysis was performed to identify the independent predictors of urinary incontinence at 6 and 12 months after RARP (Table 3). After adjusting for clinical and surgical factors, preoperative MUL was identified as a significant independent predictor of persistent incontinence at both time points. Each additional millimeter of MUL was associated with a 20% reduction in the odds of incontinence at 6 months (OR, 0.798; 95% confidence interval [CI], 0.711–0.894; *p* < 0.001) and a 41.4% reduction at 12 months (OR, 0.586; 95% CI, 0.467–0.735; *p* < 0.001). Other clinical parameters, including age (*p* = 0.412 at 6 months and *p* = 0.472 at 12 months) and operative time (*p* = 0.089 at 6 months and *p* = 0.882 at 12 months), were not statistically significant in the multivariable model. However, seminal vesicle invasion (SVI) was a significant predictor of incontinence at 6 months (OR, 3.195; 95% CI, 1.111–9.185; *p* = 0.031). Furthermore, lymph node dissection (LND) showed a trend toward an association at 6 months (OR, 2.714; 95% CI, 0.954–7.718; *p* = 0.061) and became a strongly significant predictor of incontinence at 12 months (OR, 6.994; 95% CI, 1.632–29.973; *p* = 0.009).

### 3.3. ROC Analysis and Bootstrap Validation

**Figure 2:**
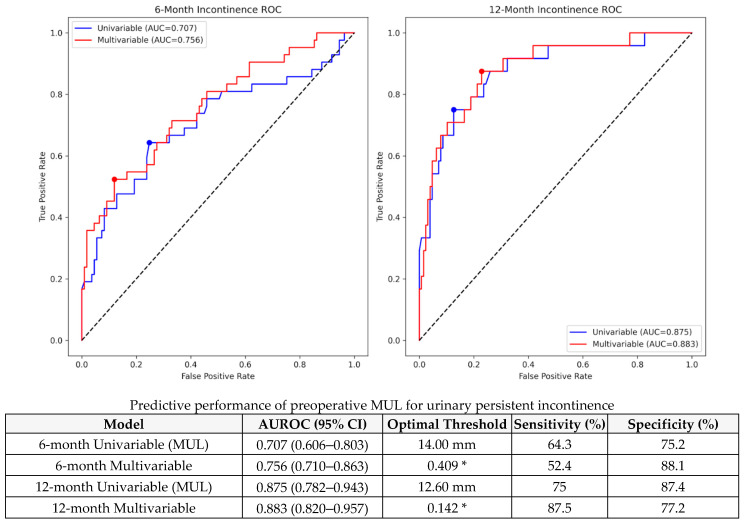
Receiver operating characteristic (ROC) curves predicting persistent incontinence at 6 months and 12 months after robot-assisted radical prostatectomy. The ROC analysis demonstrates the predictive value of preoperative membranous urethral length (MUL) for persistent incontinence. For 6-month incontinence prediction, the AUROC is 0.707 (95% confidence interval [CI], 0.606–0.803) for the univariable MUL model and 0.756 (95% CI, 0.710–0.863) for the multivariable model. For 12-month incontinence prediction, the AUROC shows excellent accuracy, with 0.875 (95% CI, 0.782–0.943) for the univariable model and 0.883 (95% CI, 0.820–0.957) for the multivariable model. The dots on the curves represent the optimal thresholds calculated using the Youden index: 14.00 mm (sensitivity 64.3%, specificity 75.2%) for the 6-month MUL and 12.60 mm (sensitivity 75%, specificity 87.4%) for the 12-month MUL. * Predicted risk probability of incontinence; lower values indicate a better prognosis for urinary continence.

The discriminative performance of MUL in predicting persistent incontinence was evaluated using ROC curve analysis (Figure 2). In the univariable analysis, MUL demonstrated fair predictive accuracy, with an AUROC of 0.707 (95% CI, 0.606–0.801) at 6 months and 0.875 (95% CI, 0.794–0.944) at 12 months, which further enhanced predictive performance, yielding AUROCs of 0.756 (95% CI, 0.698–0.874) and 0.883 (95% CI, 0.818–0.957), respectively. According to the Youden index, the optimal clinical cutoff values were determined to be 14.00 mm for 6-month persistent incontinence and 12.60 mm for 12-month persistent incontinence.

**Figure 3:**
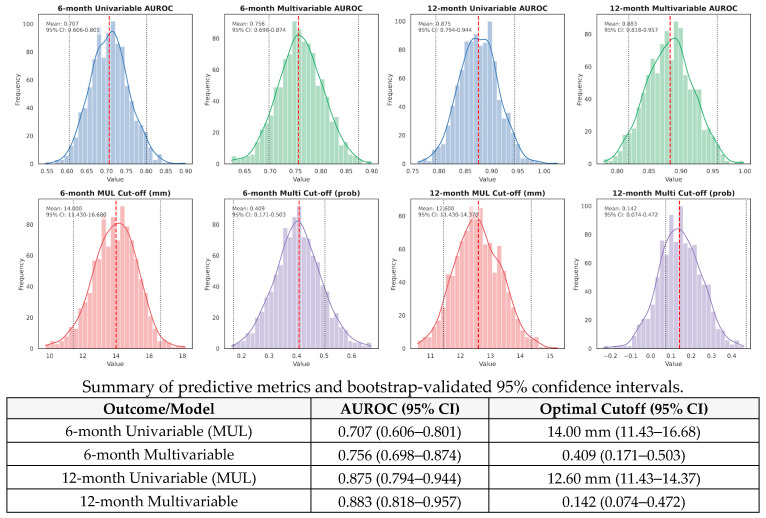
Internal validation of predictive performance and optimal thresholds using bootstrap resampling (1000 iterations). The histograms demonstrate the distributions of the area under the receiver operating characteristic curve (AUROC) and optimal cutoff values for membranous urethral length (MUL) across 1000 bootstrap iterations. For the 6-month models, the mean validated AUROC is 0.707 (95% confidence interval [CI], 0.606–0.801) for the univariable model and 0.756 (95% CI: 0.698–0.874) for the multivariable model. The corresponding optimal threshold for the 6-month MUL is 14.000 mm (95% CI, 11.430–16.680 mm). For the 12-month models, the mean validated AUROC is 0.875 (95% CI, 0.794–0.944) for the univariable model and 0.883 (95% CI, 0.818–0.957) for the multivariable model. The validated optimal threshold for a 12-month MUL is 12.600 mm (95% CI, 11.430–14.370 mm). The dashed red lines indicate the mean values of the bootstrap iterations, and the dotted black lines represent the 95% confidence intervals. AUROC, area under the receiver operating characteristic curve; CI, confidence interval; MUL, membranous urethral length. Optimal cut-off values were derived from the original dataset. 95% CIs were calculated using 1000 bootstrap resamples.

Internal validation using 1000 bootstrap iterations confirmed the stability of these thresholds across the univariable and multivariable models (Figure 3). For the univariable model, the bootstrap-validated 95% CIs for the optimal MUL cutoffs were 11.43–16.68 mm at 6 months and 11.43–14.37 mm at 12 months. Correspondingly, the validated optimal probability cutoffs for the multivariable model were 0.409 (95% CI, 0.171–0.503) at 6 months and 0.142 (95% CI, 0.074–0.472) at 12 months. These findings allow for clinical risk stratification; specifically, for 12-month outcomes, an MUL below the lower bound of the CI (<11.43 mm) indicates a higher probability of persistent incontinence, whereas an MUL above the upper bound (>14.37 mm) suggests a high recovery likelihood.

## 4. Discussion

Preoperative MUL has long been recognized as a critical anatomical determinant of PPI. Although various studies have explored this relationship, they frequently suffer from a lack of standardized measurement protocols [13]. As highlighted in recent reports, inconsistencies, such as the indiscriminate use of the sagittal and coronal planes or the subjective identification of anatomical landmarks, have introduced significant measurement variability [16].

The methodological inconsistency that the present study seeks to resolve is prominently mirrored in existing literature involving Korean cohorts. Mirroring the broader global discourse, studies focusing on Korean patients lack a unified protocol for MUL assessment. A primary source of this disparity is the absence of standardized guidelines for selecting the appropriate imaging plane (sagittal versus coronal), which constitutes a fundamental orientation benchmark for accurately tracing the urethral path. Moreover, the reliability of previous findings has been significantly undermined by substantial variability in the definition of key anatomical landmarks, such as the inferior boundary of the prostatic apex and superior aspect of the penile bulb [13,19,26,27,28,29]. In some reports, the measurement methodology was not explicitly delineated, further complicating the interpretability and reproducibility of the results [30].

Such fragmentation of criteria has led to wide variability in reported mean MUL values, presenting a significant barrier to the establishment of reliable, ethnically specific clinical thresholds in Korean men. Despite compelling evidence that Asian men possess significantly shorter MULs than non-Asian populations [22], the uncritical adoption of heterogeneous measurement techniques has failed to precisely account for the unique anatomical profile of Korean men. Furthermore, we believe that applying standardized measurement protocols to other ethnic groups is essential to establish reliable global benchmarks. Given the significant racial variations in pelvic anatomy, it is likely that these standardized assessments will yield distinct, population-specific clinical cutoff values, facilitating more accurate risk stratification across diverse populations. Consequently, to provide standardized guidelines for MUL measurement, we implemented a 3-axis CRS to minimize interobserver error and establish a more rigorous framework for MUL assessment [21]. Moving beyond mere standardization, we sought to bridge the gap between anatomical measurements and clinical applications by providing precise thresholds through a rigorous dual-method analysis, encompassing both the Youden index and 1000-iteration bootstrap validation, to determine the optimal MUL cutoffs at 6 and 12 months postoperatively.

In the present study, we derived precise cutoff values for persistent incontinence at 6 and 12 months postoperatively using the Youden index and 1000-iteration bootstrap validation. Specifically, the Youden index identified optimal thresholds of 14.00 mm and 12.60 mm at 6 and 12 months, respectively. These were closely mirrored by bootstrap validation, which yielded estimates of 14.00 mm and 12.60 mm, respectively. When synthesized for clinical utility, these values converge into reliable ranges of 11.43–16.68 mm (6 months) and 11.43–14.37 mm (12 months). This convergence across the two analytical methods underscores the internal consistency of our findings. This temporal difference in the cutoff values, specifically a higher threshold at 6 months (14.00 mm) compared to 12 months (12.60 mm), accurately reflects the dynamic mechanism of postoperative functional compensation. During the early recovery phase, continence is predominantly dependent on the immediate anatomical reserve of the preserved urethral sphincter complex, thus requiring a longer MUL to achieve continence within 6 months. However, over the subsequent months, gradual functional compensation occurs through the adaptation and strengthening of the surrounding pelvic floor musculature, such as the levator ani and external rhabdosphincter [31]. Consequently, by 12 months, this progressive muscular compensation allows patients with a relatively shorter MUL to eventually achieve continence, thoroughly explaining the lower predictive cutoff value at the 1-year mark.

These metrics extend beyond mere statistical significance and carry substantial weight in clinical decision-making. Based on the 95% CI from our bootstrap analysis, we identified critical high-risk thresholds: patients with an MUL shorter than 11.43 mm at 6 months or 11.43 mm at 12 months should be classified into a high-risk group for persistent PPI. For these individuals, surgeons must provide comprehensive preoperative counseling regarding the likelihood of prolonged incontinence. In certain cases, when considered alongside multiple oncological and patient-related factors, a short MUL may prompt a discussion of non-surgical treatment alternatives, such as radiotherapy or heavy-ion therapy, to ensure a fully informed patient choice. Conversely, an MUL exceeding 16.68 mm at 6 months or 14.37 mm at 12 months allows for a more favorable prognosis, enabling clinicians to offer patients realistic reassurance regarding early recovery. Beyond absolute cutoff values, our quartile-based analysis demonstrates a clear, stepwise improvement in persistent incontinence rates corresponding to incremental increases in preoperative MUL. Specifically, the 12-month persistent incontinence rate nearly doubled from the lowest quartile (Q1, 52.6%) to the highest quartile (Q4, 97.3%), underscoring the proportional relationship between the urethral length and functional outcomes. This stepwise stratification provides an additional layer of prognostic granularity, allowing clinicians to set more nuanced and realistic expectations for patients based on their specific MUL quartiles rather than relying solely on a binary threshold.

Beyond its role as a predictive biomarker, MUL anatomically represents the urethral lissosphincter length. While we acknowledge that macroscopic MRI has limitations in perfectly visualizing the micro-anatomical intra-apical extension of the lissosphincter, our study prioritized imaging reproducibility. To align with the philosophy of maximal urethral preservation [32,33,34], our surgical technique consistently involved maximally exposing the urethra and carefully confirming the apical boundary prior to transection. Ultimately, our reproducible MRI findings strongly support that preserving the maximal length of the lissosphincter complex is critical for optimizing postoperative continence. Furthermore, while preoperative MUL serves as a powerful baseline predictor, optimizing functional recovery ultimately depends on a comprehensive surgical approach. As emphasized in the recent literature [31], incorporating concurrent surgical maneuvers, such as the preservation of the levator fascia, puboprostatic collar, and bladder neck, alongside maintaining a long urethral stump and performing posterior reconstruction, remains essential to maximize continence outcomes.

The primary strength of this study is the systematic measurement of MUL through the implementation of a standardized 3-axis CRS, as proposed by Kwon et al. [21]. By adopting this rigorous protocol, we sought to minimize interobserver errors and ensure high data reliability. Building on the methodological foundation established in previous reports, our study represents a practical effort to transition from a purely anatomical assessment to the identification of concrete, clinically applicable thresholds. The internal validity of these findings was further confirmed through a 1000-iteration bootstrap analysis, reaffirming MUL potential as a robust preoperative predictor of PPI.

While the association between MUL and PPI has been explored in several Korean cohorts, the present study is distinguished by its focus on providing time-specific, validated cutoff intervals. Although several previous reports have provided valuable evidence that a longer MUL correlates with faster persistent incontinence in Korean men [13,28,30,35], their analyses primarily focused on identifying MUL as a significant independent variable or comparing the mean values between the continent and incontinent groups. However, from a clinical perspective, identifying that ‘longer is better’ offers limited guidance for preoperative risk stratification.

Our study advances this discourse by moving beyond general trends to determine the precise thresholds for 6-month and 12-month persistent incontinence. By utilizing the Youden index in conjunction with bootstrap-derived 95% CI, we offer a more granular framework that accounts for the unique anatomical profile of Korean men, known to have shorter MULs than Western men. Crucially, our analysis was extended beyond anatomical metrics to the development of multivariable predictive models, which demonstrated superior discriminative performance with AUROCs of 0.756 and 0.883 for 6-month and 12-month persistent incontinence, respectively (Figure 2 and Figure 3). By providing both validated MUL cutoff intervals and multivariable probability thresholds (0.409 at 6 months and 0.142 at 12 months), we established a robust clinical tool for personalized risk assessment. Although MUL remained the most robust independent predictor, our multivariable analysis also identified SVI and pelvic LND as significant factors influencing functional recovery. Notably, SVI was associated with delayed early continence at 6 months (OR 3.195), whereas LND emerged as a strong negative predictor of long-term persistent incontinence at 12 months (OR 6.994). The inclusion of these oncological and surgical variables along with MUL improved the predictive performance of our multivariable models, increasing the AUROC from 0.707 to 0.756 at 6 months and from 0.875 to 0.883 at 12 months. This highlights the necessity of adopting a comprehensive approach that integrates anatomical and surgical parameters for accurate risk assessment. This methodological rigor allows for a more nuanced classification of “high-risk” versus “low-risk” patients rather than relying on a singular, unvalidated mean value. Additionally, it should be emphasized that prostate volume was not a significant predictor of persistent incontinence in the univariable analysis at 6 months (*p* = 0.086) or 12 months (*p* = 0.408). Although a weak statistical correlation between prostate volume and MUL was observed (r = 0.266, *p* = 0.001), prostate volume typically exhibits a skewed, non-normal distribution in the clinical cohorts. Given these statistical characteristics and the lack of clinical impact, strictly treating it as a continuous variable has inherent limitations. Consequently, the observed statistical variations in prostate volume should not be overinterpreted as the primary functional determinant of persistent incontinence.

Despite the respectable AUROC values in our study, a cautious interpretation remains necessary. Importantly, the proposed MUL cutoffs were internally validated only. Because this study lacks an external validation cohort and was conducted at a single institution with a limited sample size, it inherently serves as a pilot study. Therefore, external validation in an independent cohort is strictly necessary before these thresholds can be considered broadly applicable. Furthermore, technical constraints, such as MRI resolution and the inherent subjectivity of the interpreter, mean that these specific ranges should not be applied as universal clinical standards.

However, the 95% CI derived from our analysis provides a pragmatic basis for preoperative counseling. Patients exhibiting an MUL shorter than the lower limits were statistically predisposed to a higher probability of persistent PPI. For these individuals, clinicians should consider a more intensive counseling approach, potentially discussing long-term prognosis or alternative treatment modalities. Conversely, an MUL exceeding the upper limit serves as a reliable indicator of favorable early recovery, allowing realistic preoperative reassurance.

Furthermore, comprehensive preoperative functional assessments, such as IPSS or OABSS, were available for only approximately 30% of the cohort. This is largely attributable to the clinical environment in Korea, where rapid surgical intervention is prioritized for patients with suspected prostate cancer, restricting the time for extensive functional evaluations. Additionally, the retrospective nature of the medical record review presents an inherent limitation in strictly differentiating the underlying purpose of pad usage. Specifically, it was clinically challenging to perfectly distinguish between patients using one pad solely for psychological security and those using it for actual minor urinary leakage. As noted in recent urological literature, including the comprehensive review by Rassweiler et al. [31], contemporary clinical studies often equate the use of no pads (0 pad) with the use of a safety pad (0–1 pad). Consequently, a strict 0-pad definition could not be applied, and the 0–1 pad definition was maintained to better represent real-world functional recovery in accordance with current clinical research practices.

We acknowledge that the relatively small number of events at the 12-month follow-up (24 incontinent patients) could theoretically introduce a risk of model overfitting in the multivariable analysis. However, to mitigate this risk and guarantee the statistical stability of our model, we conducted an internal validation using 1000 bootstrap resamples. This validation demonstrated consistent estimates with narrow confidence intervals, thereby confirming the reliability and robustness of our predictive model despite the limited number of outcome events.

Another limitation of this study is the potential for technique variability and selection bias. Although all surgeries were performed by two highly experienced surgeons using a standardized transperitoneal approach, inherent differences in individual surgical techniques between the two operators remain a potential confounding factor. Furthermore, the inclusion of patients requiring extended pelvic lymph node dissection implies a wider extent of surgical dissection and potentially more aggressive underlying disease states, which may have independently influenced the functional recovery trajectory. Future prospective studies should aim to meticulously control for individual surgeon variability and the extent of surgical dissection to further validate our findings.

Finally, there was no standardized pelvic floor muscle training or rehabilitation program linked to postoperative care at our institution during the study period. Therefore, patients were not uniformly instructed to perform Kegel exercises, and we could not assess or control individual adherence. Because postoperative rehabilitation may independently influence the trajectory of continence recovery, the inability to account for this variable represents another limitation of our study.

As more high-quality data from well-designed studies accumulate, we anticipate that these CI ranges will be further refined, leading to more definitive clinical criteria. In conclusion, MUL is an independent and powerful predictor of persistent incontinence after RARP. To overcome the limitations of this pilot study, future multicenter studies are essential to establish national standard cutoff values. Furthermore, the development of artificial intelligence-driven automated CRS measurement systems is a pivotal step toward enhancing the objectivity and clinical reproducibility of MUL assessment.

## 5. Conclusions

In conclusion, preoperative MUL, assessed using a highly reproducible three-axis CRS, is a robust and independent predictor of urinary persistent incontinence following RARP in Korean men. Supported by internal validation using a 1000-iteration bootstrap analysis, our findings demonstrate that a longer urethral length is consistently associated with a significantly reduced risk of incontinence throughout the first postoperative year. By providing time-specific, validated risk intervals tailored to the unique anatomical profile of the Korean population, this study transcends general anatomical observations and offers a pragmatic framework for preoperative risk stratification. These precise data will enable clinicians to provide more personalized counseling, realistically manage patient expectations, and refine treatment strategies for patients identified as being at a higher risk of persistent incontinence, ultimately facilitating better-informed clinical decision-making.

## Figures and Tables

**Table 1:** Baseline characteristics.

Variable	6-Month			12-Month			Total Cohort
	Continence	Incontinence	*p*	Continence	Incontinence	*p*	
Patients (*n*)	109	42		127	24		151
Age (years)	69.5 ± 6.7	69.9 ± 8.0	0.797	69.5 ± 6.7	69.9 ± 9.2	0.868	69.6 ± 7.1
BMI (kg/m^2^)	25.3 ± 2.9	24.4 ± 2.5	0.085	25.2 ± 2.9	24.4 ± 2.5	0.181	25.0 ± 2.8
Initial PSA (ng/mL)	10.8 ± 13.2	13.5 ± 10.9	0.196	11.3 ± 13.2	12.4 ± 9.0	0.629	11.5 ± 12.6
Prostate volume (mL)	39.4 ± 12.7	43.5 ± 13.2	0.086	40.9 ± 13.6	38.5 ± 8.6	0.268	40.5 ± 13.0
MUL (mm)	16.7 ± 3.7	13.7 ± 4.4	<0.001	16.7 ± 3.7	11.3 ± 3.1	<0.001	15.9 ± 4.1
Operative time (min)	155.0 ± 37.4	176.7 ± 38.9	0.003	158.8 ± 39.4	172.8 ± 34.6	0.084	161.1 ± 38.9
Estimated blood loss (mL)	226.8 ± 178.6	221.9 ± 165.6	0.874	231.3 ± 177.2	194.6 ± 159.6	0.318	225.4 ± 174.6
Hypertension	65 (59.6%)	22 (52.4%)	0.532	74 (58.3%)	13 (54.2%)	0.883	87 (57.6%)
Diabetes mellitus	25 (22.9%)	8 (19.0%)	0.765	29 (22.8%)	4 (16.7%)	0.688	33 (21.9%)
Seminal vesicle invasion	12 (11.0%)	13 (31.0%)	0.007	21 (16.5%)	4 (16.7%)	0.999	25 (16.6%)
Extracapsular invasion	25 (22.9%)	16 (38.1%)	0.094	33 (26.0%)	8 (33.3%)	0.623	41 (27.2%)
Positive surgical margin	32 (29.4%)	14 (33.3%)	0.781	38 (29.9%)	8 (33.3%)	0.927	46 (30.5%)
Lymph node dissection	13 (11.9%)	17 (40.5%)	<0.001	19 (15.0%)	11 (45.8%)	0.001	30 (19.9%)
None	96 (88.1%)	25 (59.5%)		108 (85.0%)	13 (54.2%)		121 (80.1%)
Unilateral	6 (5.5%)	0 (0.0%)		6 (4.7%)	0 (0.0%)		6 (4.0%)
Bilateral	7 (6.4%)	17 (40.5%)		13 (10.2%)	11 (45.8%)		24 (15.9%)
Nerve-sparing	106 (97.2%)	40 (95.2%)	0.211	124 (97.6%)	22 (91.7%)	0.133	146 (96.7%)
None	3 (2.8%)	2 (4.8%)		3 (2.4%)	2 (8.3%)		5 (3.3%)
Unilateral	5 (4.6%)	5 (11.9%)		7 (5.5%)	3 (12.5%)		10 (6.6%)
Bilateral	101 (92.7%)	35 (83.3%)		117 (92.1%)	19 (79.2%)		136 (90.1%)
Gleason Score			0.089			0.789	
≤6	18 (16.5%)	7 (16.7%)		20 (15.7%)	5 (20.8%)		25 (16.6%)
7	70 (64.2%)	20 (47.6%)		77 (60.6%)	13 (54.2%)		90 (59.6%)
≥8	21 (19.3%)	15 (35.7%)		30 (23.6%)	6 (25.0%)		36 (23.8%)

**Table 2:** Postoperative urinary continence rates stratified by preoperative membranous urethral length (MUL) quartiles.

MUL Quartile	*n*	MUL, Median (Range), mm	Continence at 6 Months, *n* (%)	Continence at 12 Months, *n* (%)
Q1	38	11.0 (6.3–13.2)	18 (47.4%)	20 (52.6%)
Q2	38	14.2 (13.3–15.8)	28 (73.7%)	34 (89.5%)
Q3	38	17.3 (15.8–18.5)	33 (86.8%)	37 (97.4%)
Q4	37	21.1 (18.6–28.2)	30 (81.1%)	36 (97.3%)

Note: For a detailed comparison of baseline clinical and pathological parameters across the MUL quartiles, please refer to Supplementary Table S1.

**Table 3:** Multivariable logistic regression analysis for independent predictors of urinary incontinence at 6 and 12 months after RARP.

Variable	OR [95% CI] at 6 Months	*p*	OR [95% CI] at 12 Months	*p*
MUL (mm)	0.798 [0.711–0.894]	<0.001	0.586 [0.467–0.735]	<0.001
Age (year)	1.025 [0.967–1.086]	0.412	1.03 [0.951–1.115]	0.472
LND *	2.714 [0.954–7.718]	0.061	6.994 [1.632–29.973]	0.009
Operative time (min)	1.01 [0.998–1.022]	0.089	1.001 [0.983–1.021]	0.882
SVI	3.195 [1.111–9.185]	0.031	0.767 [0.148–3.981]	0.752

OR, odds ratio; CI, confidence interval; MUL, membranous urethral length; LND, pelvic lymph node dissection; SVI, seminal vesicle invasion. * LND and NS were categorized as “performed” if the procedure was conducted either unilaterally or bilaterally. Note: For the comprehensive univariable analysis that informed this multivariable model, please refer to the Supplementary Table S2.

## Data Availability

The data presented in this study are available on request from the corresponding author. The data are not publicly available due to privacy and ethical restrictions.

## Supplementary Materials

The following supporting information can be downloaded at: https://www.mdpi.com/article/10.3390/jcm15124454/s1, Figure S1: Scatter plot demonstrating the correlation between prostate volume and membranous urethral length; Table S1: Clinical and pathologic characteristics across the MUL quartiles; Table S2: Univariable logistic regression analysis predicting incontinence recovery at 6 and 12 months postoperatively.

## Abbreviations

The following abbreviations are used in this manuscript:

AUROC	Area under the receiver operating characteristic curve
BMI	Body mass index
CI	Confidence interval
CRS	Cross-reference system
IRB	Institutional Review Board
LND	Lymph node dissection
mpMRI	Multiparametric magnetic resonance imaging
MUL	Membranous urethral length
NS	Nerve-sparing
OR	Odds ratio
PI-RADS	Prostate Imaging-Reporting and Data System
PPI	Post-prostatectomy urinary incontinence
PSA	Prostate-specific antigen
RARP	Robot-assisted radical prostatectomy
ROC	Receiver operating characteristic
SVI	Seminal vesicle invasion
