# Supplementary material for: Refining the Predictive Accuracy of Membranous Urethral Length for Post-Prostatectomy Incontinence: A Standardized Approach in the Korean Population
Source: J Clin Med. 2026 Jun 9;15(12):4454. doi: 10.3390/jcm15124454 (PMC13301952; doi:10.3390/jcm15124454)
Supplement: Supplementary file 1 [file jcm-15-04454-s001.zip › jcm-4319337-supplementary.pdf]

Supplementary Table S1. Clinical and pathological characteristics across the MUL quartiles

Variable	Q1	Q2	Q3	Q4	*p*-value
Patients (n)	38	38	38	37	
Age (years)	69.3 ± 8.8	69.4 ± 5.9	69.0 ± 6.6	70.8 ± 6.9	0.7
BMI (kg/m2)	25.1 ± 2.8	24.5 ± 2.3	25.2 ± 3.0	25.4 ± 3.1	0.526
Initial PSA (ng/mL)	10.9 ± 7.2	10.3 ± 9.2	10.9 ± 11.4	14.1 ± 19.5	0.561
Prostate volume (mL)	37.7 ± 9.0	37.7 ± 11.0	40.5 ± 12.1	46.4 ± 17.1	0.009
MUL (mm)	10.7 ± 1.6	14.3 ± 0.8	17.4 ± 0.8	21.2 ± 2.0	< 0.001
Operative time (min)	165.9 ± 35.3	154.8 ± 40.1	163.8 ± 42.4	159.7 ± 38.2	0.624
Estimated blood loss (mL)	203.4 ± 159.2	223.2 ± 156.1	219.2 ± 190.7	256.8 ± 192.3	0.608
Hypertension	19 (50.0%)	26 (68.4%)	20 (52.6%)	22 (59.5%)	0.368
Diabetes mellitus	8 (21.1%)	5 (13.2%)	7 (18.4%)	13 (35.1%)	0.123
Seminal vesicle invasion	5 (13.2%)	7 (18.4%)	7 (18.4%)	6 (16.2%)	0.916
Extracapsular invasion	12 (31.6%)	9 (23.7%)	10 (26.3%)	10 (27.0%)	0.892
Positive surgical margin	13 (34.2%)	13 (34.2%)	10 (26.3%)	10 (27.0%)	0.797
Lymph node dissection					0.585
None	29 (76.3%)	29 (76.3%)	33 (86.8%)	30 (81.1%)	
Unilateral	2 (5.3%)	3 (7.9%)	1 (2.6%)	0 (0.0%)	
Bilateral	7 (18.4%)	6 (15.8%)	4 (10.5%)	7 (18.9%)	
Nerve-sparing					0.984
None	1 (2.6%)	2 (5.3%)	1 (2.6%)	1 (2.7%)	
Unilateral	2 (5.3%)	2 (5.3%)	3 (7.9%)	3 (8.1%)	
Bilateral	35 (92.1%)	34 (89.5%)	34 (89.5%)	33 (89.2%)	
Gleason Score					0.318
≤ 6	6 (15.8%)	6 (15.8%)	10 (26.3%)	3 (8.1%)	
7	22 (57.9%)	26 (68.4%)	20 (52.6%)	22 (59.5%)	
≥ 8	10 (26.3%)	6 (15.8%)	8 (21.1%)	12 (32.4%)	

Supplementary Table S2. Univariable logistic regression analysis predicting persistent incontinence at 6 and 12 months postoperatively

Variable	6-month			12-month		
	OR	95% CI	*p*	OR	95% CI	*p*
Age (years)	1.007	0.958–1.060	0.778	1.007	0.946–1.071	0.833
BMI (kg/m2)	0.895	0.781–1.026	0.111	0.9	0.763–1.063	0.215
Initial PSA (ng/mL)	1.016	0.989–1.043	0.257	1.006	0.975–1.038	0.706
Prostate volume (mL)	1.023	0.997–1.051	0.086	0.984	0.947–1.022	0.408
MUL (mm)	0.821	0.740–0.910	< 0.001	0.606	0.495–0.741	< 0.001
Operative time (min)	1.014	1.005–1.024	0.003	1.009	0.998–1.020	0.11
Estimated blood loss (mL)	1.000	0.998–1.002	0.877	0.999	0.996–1.001	0.346
Hypertension	0.745	0.364–1.524	0.42	0.846	0.352–2.035	0.709
Diabetes mellitus	0.791	0.325–1.926	0.605	0.676	0.214–2.136	0.505
Seminal vesicle invasion	3.624	1.492–8.802	0.004	1.01	0.313–3.256	0.987
Extra capsular invasion	2.068	0.961–4.449	0.063	1.424	0.558–3.634	0.459
Positive surgical margin	1.203	0.561–2.579	0.635	1.171	0.462–2.967	0.739
Lymph node dissection (LND)						
None (reference)						
Unilateral	N/A*	N/A*	N/A*	N/A*	N/A*	N/A*
Bilateral	9.326	3.486–24.950	< 0.001	7.03	2.618–18.877	< 0.001
Nerve-sparing						
None	1.924	0.309–11.993	0.483	4.105	0.643–26.206	0.135
Unilateral	2.886	0.788–10.566	0.109	2.639	0.627–11.103	0.186
Bilateral (reference)						
Gleason Score						
≤ 6 (reference)						
7	0.735	0.269–2.006	0.547	0.675	0.215–2.117	0.501
≥ 8	1.837	0.614–5.496	0.277	0.8	0.215–2.979	0.739

*N/A: Not applicable due to zero events of incontinence in the unilateral LND group

Supplementary Figure S1. Scatter plot demonstrating the correlation between prostate volume and membranous urethral length

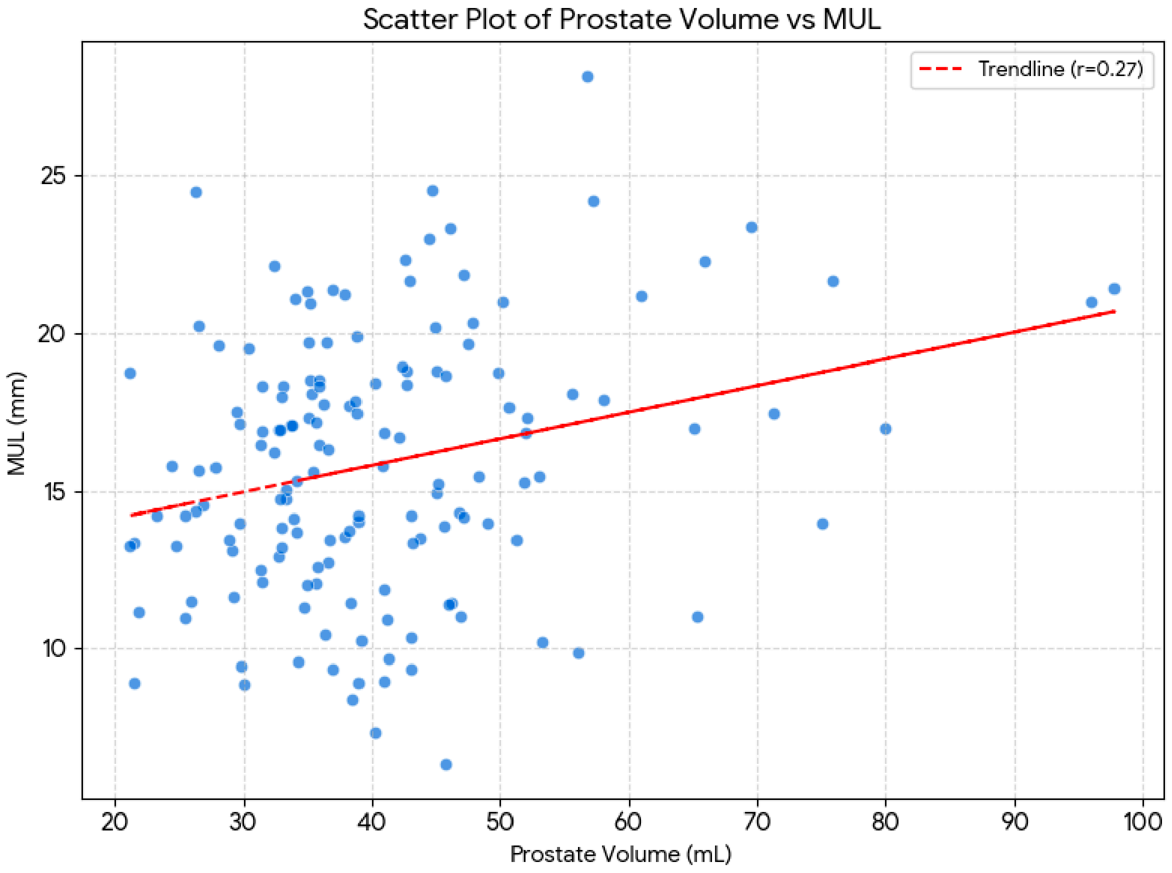

The scatter plot illustrates the anatomical relationship between prostate volume (mL) and membranous urethral length (MUL, mm) measured on preoperative magnetic resonance imaging. Although statistically significant, a weak positive correlation is observed (Pearson's correlation coefficient, r = 0.266, p = 0.001). The dashed line represents the linear trend. Although prostate volume exhibits a statistically significant correlation with MUL, the magnitude of this association is weak. Furthermore, prostate volume is not a significant predictor of persistent incontinence in either the comparative analysis between continence groups (Table 1) or the univariable logistic regression analysis (Supplementary Table S2).
